# A meta-analysis on the EBV DNA and VCA-IgA in diagnosis of Nasopharyngeal Carcinoma

**DOI:** 10.12669/pjms.293.2907

**Published:** 2013

**Authors:** Changxin Song, Shujuan Yang

**Affiliations:** 1 Changxin Song, Department of Computer, Qinghai Normal University, Xining, China.; 2 Shujuan Yang, West China School of Public Health, Sichuan University, Chengdu, China.

**Keywords:** Epstein-Barr virus (EBV), DNA, VCA-IgA, Diagnosis, Nasopharyngeal Carcinoma

## Abstract

***Objective:*** We conducted a meta-analysis to compare the EBV DNA and VCA-IgA in diagnosis of Nasopharyngeal Carcinoma, and provide important evidence for screening method of NPC.

***Methodology:*** Three databases, Medline (from Jan. 1966 to Jan. 2012), EMBASE (from January 1988 to Jan. 2012) and Chinese Biomedical Database (from January 1980 to Jan. 2012) were used to detect the role of EBV DNA and VCA-IgA in diagnosis of NPC. Meta-DiSc statistical software was used for analysis.

***Results:*** Twenty seven case-control and cohort studies were included in final analysis. A total of 1554 cases and 2932 controls were included in our meta-analysis. The Sensitivity specificity, positive likelihood (+LR) and likelihood negative (-LR) of EBV-DNA in diagnosis of NPC were 0.75(0.72-0.76), 0.87(0.85-0.88), 6.98(4.50-10.83) and 0.18(0.11-0.29), respectively, and they were 0.83(0.81-0.85), 0.85(0.83-0.86), 10.89(5.41-21.93) and 0.20(0.14-0.29) for VCA-IgA. The SROC for EBV DNA detection was 0.939, while this was 0.936 for VCA-IgA detection. The subgroup analysis showed EBV-DNA had larger areas under the summary receiver operator curve when compared with VCA-IgA in high quality and low quality studies.

***Conclusion:*** Our meta-analysis indicated the EBV DNA had higher sensitivity and specificity in diagnosis of NPC.

## INTRODUCTION

Nasopharyngeal carcinoma (NPC) is a rare disease on a world scale, and it accounted for 0.7% of all cancers, and ranked the 23rd most common new cancer in the world.^1^ However, it is endemic in some specific areas, such as in Hong Kong, and south of China.^1^ The intermediate rates are observed in several indigenous populations in South East Asia and in natives of the Arctic region, North Africa and the Middle East.^1^ Epstein-Barr Virus (EBV) is a well known risk factor for NPC. Patients with NPC are noted to have high levels of EBV antibodies.^2^^,^^3^ The infection of EBV is not associated directly in inducing by the tumor, but infection in the healthy individuals means increased risk of cancer.^4^^-^^6^

Several diagnostic methods are used for NPC detection, but the EBV serology examinations test IgA antibodies against viral capsid antigen (VCA) to IgA to early antigen are the most common detection methods for diagnosis of NPC.^2^ This method is cheap and non- invasive, and therefore, it is acceptable for patients and could be widely used in clinics. Quantitative EBV DNA and VCA-IgA analysis has been reported to be a sensitive detection tool in diagnosis of NPC.^7^^,^^8^

Recent studies indicated the cell-free EBV DNA had high detection rate in the plasma and serum among patients with NPC.^9^^,^^10^ Recently several studies have showed plasma EBV-DNA and VCA-IgA level might be a sensitive and reliable biomarker for the diagnosis of NPC at a molecular level in clinical practice.^11^^-^^14^ However, the there is no consensus yet which is a better test for the early diagnosis of nasopharyngeal carcinoma. Reasons may include the different sources of EBV antigens, different antibody assays and the selection of cases from different geographic origins. Therefore, we conducted a meta-analysis to evaluate which EBV serology examination had the better sensitivity and specificity in the diagnosis of NPC.

## METHODOLOGY

***Searching strategy: ***Three databases, Medline (from Jan. 1966 to Jan. 2012), EMBASE (from January 1988 to Jan. 2012) and Chinese Biomedical Database (from January 1980 to Jan. 2012), were systematically searched by using related terms (‘Epstein-Barr Virus’, ‘EBV’, ‘DNA’, ‘VCA-IgA’, ‘serological test’, ‘nasopharyngeal carcinoma’ (NPC). There was no restriction on the language of the papers. References cited in retrieved studies were reviewed for more eligible studies. The criteria used for including studies were (1) Case-control or cohort studies on the role of EBV-DNA and VCA-IgA in diagnosis of NPC; (2) identification of NPC was confirmed histologically/pathologically; (3) Available data regarding sensitivity and specificity of EBV-DNA and VCA-IgA in diagnosis of NPC; If the authors reported more than once the data on publication papers, we only included the complete data into our review. The exclusion criteria were case only study, reviews, and overlapping studies.

***Data extraction: ***Two reviewers independently reviewed the final abstracts of all potential articles, and decided one should be included into final meta-analysis. In case there was any disagreement, it was resolved by discussion. If the data were missing in the included studies, we attempted to contact the authors by emails or telephones in order to include complete data. From these finally selected studies, we included author’s names, location, study type, number of participants of studies in terms of EBV-DNA and VCA-IgA (Table-I).

***Quality of study: ***The quality of included studies was according to the Cochrane Handbook for diagnostic test accuracy review. The criteria included sampling, data collection, design of study, detection application and selection bias. The quality scores ranged from 0 to 10. Score<6 was defined as low quality, and score≥6 was defined as high quality.

***Statistical analysis: ***Statistical analysis was conducted by using Meta-DiSc statistical software version 1.4 (Unit of Clinical Biostatistics, Ramony Cajal Hospital, Madrid, Spain). The accuracy indexes of EBV-DNA and VCA-IgA was pooled by meta-analysis, such as sensitivity, specificity, positive likelihood ratio (LR+) and negative likelihood ratio (LR–). The heterogeneity was evaluated by I^2^ with p-values < 0.1. The I^2^ value of 25%, 50% and 75% were regarded as low, moderate and high heterogeneity, respectively (Higgins et al., 2003)^39^ and its possible sources of heterogeneity were evaluated by subgroup analysis. If moderate or high heterogeneity existed, the random effects model was used. Otherwise, a fixed-effect model was used for pooled results. Summary receiver operating characteristic (SROC) curve was used for evaluating the global summary of test performance, and the area under the SROC curve presents the overall performance of the detection method. The area under the curve of 1 presents perfect discriminatory ability. All P values are two sides and P<0.05 was regarded as statistical significant.

## RESULTS

***Characteristics of studies:*** A total of 758 records were selected by searching the databases. After excluding the overlapping studies and those which were not in line with the inclusion criteria. A total of 29 studies were included and assessed for meta-analysis. After reviewing the original paper, we excluded 2 studies. Finally, 27 case-control and cohort studies were included in final analysis. A total of 2717 cases and 4085 controls were included in our meta-analysis (Table-I).

**Table-I:** Characteristics of included studies

*Study ID*	*Location*	*Sample size*		*Method*	*Study design*	*Score of bias*
		*Case*	*Control*			
Zhang 2012[15]	Mainland China	40	50	EBA DNA	Case-control	6
Zhu 2012[16]	Mainland China	168	60	EBA DNA and VCA-IgA	Case-control	6
Feng 2009 [17]	Mainland China	65	29	EBA DNA and VCA-IgA	Case-control	7
Kong 2010 [18]	Mainland China	56	60	EBA DNA	Case-control	9
Liao 2010 [19]	Mainland China	34	30	EBA DNA and VCA-IgA	Case-control	4
Sun 2010 [20]	Mainland China	62	62	EBA DNA and VCA-IgA	Case-control	5
Tan 2010[21]	Mainland China	12	40	EBA DNA and VCA-IgA	Case-control	3
Wai 2010[22]	Hong Kong	18	1181	EBA DNA	Case-control	8
Luo 2009 [23]	Mainland China	160	76	EBA DNA and VCA-IgA	Case-control	5
Chang 2008 [24]	Mainland China	156	265	EBA DNA	Cohort	5
Sun 2008[25]	Mainland China	68	90	EBA DNA and VCA-IgA	Case-control	5
Ozyar 2007[26]	Turkey	24	29	EBA DNA	Case-control	7
O 2007[27]	United State	24	84	EBA DNA and VCA-IgA	Case-control	8
Li 2007[28]	China	781	171	VCA-IgA	Case-control	5
Huang 2006[29]	China	184	80	VCA-IgA	Case-control	5
Leung 2004 [30]	Hong Kong	139	178	EBA DNA and VCA-IgA	Case-control	7
Shao 2004[31]	Mainland China	147	78	EBA DNA	Case-control	6
Fan 2004[32]	Mainland China	65	68	EBA DNA and VCA-IgA	Case-control	5
Krishna 2004[33]	India	17	15	EBA DNA	Case-control	6
Chan 2003[34]	Mainland China	55	163	EBA DNA and VCA-IgA	Case-control	5
Pratesi 2003[35]	Italy	15	32	EBA DNA	Case-control	7
Fang 2003[36]	China	114	842	VCA-IgA	Case-control	5
Huang 2003[37]	China	84	60	VCA-IgA	Case-control	4
Mai 2002[38]	Mainland China	66	58	EBA DNA	Case-control	5
Mutirangura 1998[8]	Thailand	13	111	EBA DNA	Case-control	7
Shotelersuk 2000[39]	Thailand	93	130	EBA DNA	Case-control	6
Lo 1999[9]	Hong Kong	57	43	EBA DNA	Case-control	8
Total		1554	2932			

**Table-II:** The diagnostic characteristics of included studies in terms of EBV-DNA

*Study ID*	*TP*	*FP*	*FN*	*TN*	*Sensitivity(95% CI)*	*Specificity(95% CI)*	*+LR(95% CI)*	*-LR(95% CI)*
Zhang 2012	27	10	13	40	0.68(0.51-0.81)	0.80(0.66-0.90)	3.38(1.86-6.12)	0.41(0.26-0.65)
Zhu 2012	58	2	110	58	0.35(0.27-0.42)	0.97(0.88-1.0)	10.36(2.61-41.1)	0.68(0.60-0.76)
Kong 2010	41	7	15	53	0.73(0.60-0.84)	0.88(0.77-0.95)	6.28(2.07-12.82)	0.30(0.20-0.47)
Liao 2010	20	3	14	27	0.59(0.41-0.75)	0.90(0.74-0.98)	5.88(1.94-17.85)	0.46(0.30-0.70)
Sun 2010	59	4	3	58	0.94(0.86-0.99)	0.94(0.84-0.98)	14.75(5.71-38.12)	0.05(0.02-0.16)
Tan 2010	33	0	90	40	0.27(0.91-1.0)	1.00(0.91-1.0)	22.15(1.39-353.54)	0.74(0.66-0.83)
Wai 2010	15	153	3	1028	0.83(0.57-0.96)	0.87(0.85-0.89)	6.43(4.99-8.29)	0.19(0.07-0.54)
Feng 2009	45	1	20	28	0.69(0.57-0.80)	0.97(0.82-0.99)	20.08(2.91-138.69)	0.32(0.22-0.46)
Luo 2009	110	9	50	67	0.69(0.61-0.76)	0.88(0.79-0.94)	5.81(3.12-10.82)	0.35(0.28-0.45)
Chang 2008	127	9	29	255	0.81(0.74-0.87)	0.97(0.94-0.98)	23.88(12.51-45.58)	0.19(0.14-0.27)
Sun 2008	65	6	3	84	0.96(0.88-0.99)	0.93(0.86-0.98)	14.34(6.61-31.11)	0.05(0.02-0.14)
Ozyar 2007	24	10	0	19	1.00(0.86-1.00)	0.66(0.46-0.82)	2.8(1.71-4.57)	0.03(0.01-0.48)
O 2007	17	7	5	79	0.77(0.55-0.92)	0.92(0.84-0.97)	9.49(4.51-20.0)	0.25(0.11-0.54)
Leung 2004	132	4	7	174	0.95(0.90-0.98)	0.98(0.94-0.99)	42.26(16.02-111.44)	0.05(0.03-0.11)
Fan 2004	64	29	1	39	0.99(0.92-1.0)	0.57(0.45-0.69)	2.31(1.75-3.05)	0.03(0.01-0.19)
Shao 2004	138	12	9	66	0.94(0.88-0.97)	0.85(0.75-0.92)	6.10(3.62-10.29)	0.07(0.04-0.14)
Krishna 2004	15	2	5	10	0.75(0.51-0.91)	0.83(0.52-0.98)	4.5(1.24-16.35)	0.3(0.14-0.67)
Chan 2003	31	3	24	160	0.56(0.42-0.70)	0.98(0.95-0.99)	30.62(9.75-96.23)	0.45(0.33-0.60)
Pratesi 2003	15	20	0	12	1.0(0.78-1.0)	0.38(0.21-0.56)	1.56(1.18-2.07)	0.08(0.01-1.31)
Mai 2002	56	6	10	52	0.85(0.74-0.93)	0.90(0.79-0.96)	8.20(3.82-17.62)	0.17(0.10-0.30)
Mutirangura 1998	13	29	0	82	1.0(0.75-0.82)	0.74(0.65-0.82)	3.66(2.64-5.07)	0.05(0.003-0.74)
Shotelersuk 2000	83	63	10	67	0.89(0.81-0.94)	0.52(0.43-0.60)	1.84(1.52-2.23)	0.21(0.11-0.38)
Lo 1999	55	3	2	40	0.97(0.88-0.95)	0.93(0.81-0.99)	13.83(4.64-41.24)	0.04(0.01-0.15)
Pooled results	1243	392	423	2538	0.75(0.72-0.76)	0.87(0.85-0.88)	6.98(4.50-10.83)	0.18(0.11-0.29)

**Table-III:** The diagnostic characteristics of included studies in terms of VCA-IgA

*Study ID*	*TP*	*FP*	*FN*	*TN*	*Sensitivity*	*Specificity*	*+LR(95% CI)*	*-LR(95% CI)*
Zhu 2012	105	2	53	28	0.67(0.58-0.74)	0.93(0.78-0.99)	9.97(2.60-38.20)	0.36(0.28-0.46)
Liao 2010	15	1	19	29	0.44(0.27-0.61)	0.97(0.83-0.99)	13.24(1.86-94.32)	0.58(0.43-0.79)
Sun 2010	58	32	5	58	0.92(0.82-0.97)	0.64(0.54-0.74)	2.59(1.94-3.45)	0.12(0.05-0.29)
Tan 2010	88	1	35	39	0.72(0.63-0.79)	0.98(0.87-1.0)	28.62(4.12-198.85)	0.29(0.22-0.39)
Luo 2009	120	4	40	72	0.75(0.68-0.82)	0.95(0.87-0.89)	14.25(5.47-37.14)	0.26(0.20-0.35)
Sun 2008	15	153	3	1028	0.83(0.59-0.96)	0.87(0.85-0.89)	6.43(4.99-8.29)	0.19(0.07-0.54)
O 2007	29	57	3	66	0.91(0.75-0.98)	0.54(0.44-0.63)	1.96(1.57-2.44)	0.18(0.06-0.52)
Li 2007	704	0	77	171	0.90(0.88-0.92)	1.0(0.98-1.0)	309.91(19.46-4935.2)	0.10(0.08-0.12)
Huang 2006	146	2	38	78	0.79(0.73-0.85)	0.98(0.91-0.99)	31.74(8.06-125.96)	0.21(0.16-0.28)
Leung 2004	112	8	27	170	0.81(0.73-0.87)	0.96(0.91-0.98)	17.93(9.06-35.46)	0.20(0.15-0.29)
Chan 2003	40	5	4	94	0.91(0.78-0.76)	0.95(0.89-0.98)	18.0(7.62-42.50)	0.10(0.04-0.24)
Fang 2003	107	193	7	649	0.94(0.88-0.96)	0.77(0.74-0.80)	4.10(3.59-4.68)	0.08(0.04-0.16)
Huang 2003	146	2	38	78	0.79(0.73-0.85)	0.98(0.91-1.0)	31.74(8.06-124.96)	0.21(0.16-0.28)
Pooled results	1685	460	349	2560	0.83(0.81-0.85)	0.85(0.83-0.86)	10.89(5.41-21.93)	0.20(0.14-0.29)

**Table-IV:** The diagnostic characteristics of EBV DNA in plasma and serum

*Subgroup*	*TP*	*FP*	*FN*	*TN*	*Pooled Sensitivity*	*Pooled Specificity*	*Pooled +LR(95% CI)*	*Pooled -LR(95% CI)*	*SROC*
*High quality of studies*									
EBV-DNA	678	323	199	1756	0.77(0.74-0.80)	0.85(0.83-0.86)	5.54(2.25-9.16)	0.16(0.07-0.37)	0.93
VCA-IgA	246	67	83	264	0.75(0.69-0.79)	0.80(0.75-0.84)	6.94(0.43-111.52)	0.25(0.13-0.47)	0.89
*Low qu* *ality of studies*									
EBV-DNA	565	69	224	782	0.72(0.68-0.75)	0.92(0.90-0.94)	10.20(4.27-24.36)	0.20(0.09-0.43)	0.96
VCA-IgA	1439	393	266	2296	0.84(0.82-0.86)	0.85(0.84-0.87)	13.05(5.69-29.93)	0.19(0.12-0.29)	0.944

**Fig.1:**
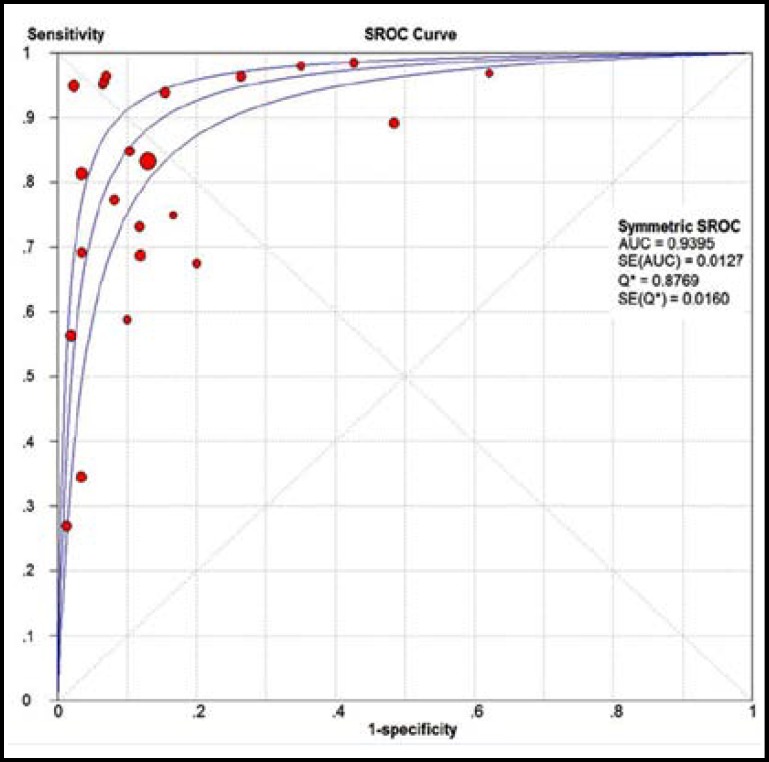
SROC for the pooled accuracy of EBV-DNA for NPC detection

**Fig.2:**
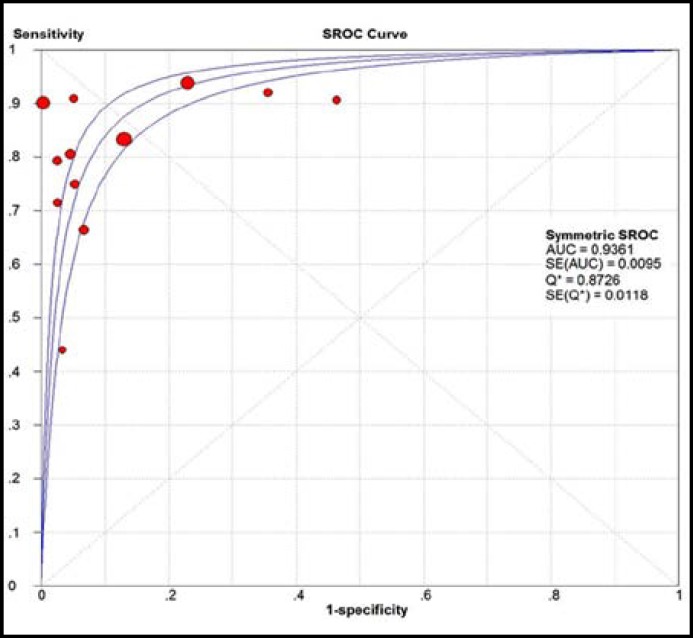
SROC for the pooled accuracy of VCA-IgA for NPC detection

We analyzed the pooled sensitivity, specificity, positive likelihood (+LR) and likelihood negative (-LR) of EBV-DNA and VCA-IgA (Table II and III). The Sensitivity specificity, positive likelihood (+LR) and likelihood negative (-LR) of EBV-DNA in diagnosis of NPC were 0.75(0.72-0.76), 0.87(0.85-0.88), 6.98(4.50-10.83) and 0.18(0.11-0.29), respectively, and they were 0.83(0.81-0.85), 0.85(0.83-0.86), 10.89(5.41-21.93) and 0.20(0.14-0.29) for VCA-IgA.

The largest area of diagnosis under the summary receiver operator curve (AUC) for NPC by overall EBV DNA detection was 0.939, while the SROC was 0.936 for VCA-IgA detection (Fig. 1 and 2). In the pooled analysis for EBV-DNA, there was significant heterogeneity across studies (p<0.05, I^2^>50%). While, no significant heterogeneity was found between studies in terms of VCA-IgA.

Subgroup analysis was taken according to the quality of studies to investigate the heterogeneity within the included studies (Table-IV), which indicated studies with low quality had lower sensitivity, specificity, +LR and -LR for both EBV-DNA and VCA-IgA detection. We could find the EBV-DNA had larger areas under the summary receiver operator curve when compared with VCA-IgA in high quality and low quality studies. The subgroup analysis significantly decreases the heterogeneity among studies, with the p value of 0.12 for EBV-DNA and 0.31 for VCA-IgA methods.

A single study in our meta-analysis was removed each time to analyze the robust of the pooled results, and the results did not greatly changed (Data not shown). The Egger’s test were used to assess the publication bias, and no significant publication bias was found in our meta-analysis.

## DISCUSSION

Meta-analysis has been regarded as an important tool to more precisely define the effect of treatment for diseases and to identify potentially important sources of between-study heterogeneity. There is no systematic review to compare the EBV DNA and VCA-IgA in diagnosis of NPC. Only one previous study showed the sensitivity and specificity of EBV DNA in diagnosis of NPC^38^, but it could not reach a conclusive result whether EBV DNA is better for VCA-IgA. Hence, our study included 27 recently published studies comparing the effectiveness EBV DNA and VCA-IgA in diagnosis of NPC. Our meta-analysis involved 2757 cases and 4085 controls. Finally, we found EBV DNA had a higher accuracy than VCA-IgA in diagnosis of NPC. The EBV DNA had large SROC of 0.94, while the VCA-IgA had SROC of 0.936. Morever, the high quality of studies in terms of EBV DNA detection had high accuracy in diagnosis of NPC when compared with VCA-IgA (AUC of EBV DNA: 0.93; AUC of VCA-IgA: 0.89).

Heterogeneity is a potential problem in explaining the results of meta-analysis, and identifying the sources of heterogeneity is an important goals of meta-analysis.^39^ In our study, we assessed the between-study heterogeneity by using the I2 statistic to quantify the between-study heterogeneity^39^, and the results suggested great heterogeneity between studies in terms of EBV-DNA. Therefore, we further performed subgroup analysis by risk of bias. The results showed that risk of bias was an main source of heterogeneity.

There are two possible limitations in our meta-analysis which mainly influence the explanation of the results. Firstly, there might be publication bias in our study. All the studies included into meta-analysis were published paper; however, there might be many unfavorable results which may not have been published. We plan to include more studies in clinical trials registration and paper presented in conferences. Secondly, there might be selection bias in our study. Secondly as most of the studies included the NPC cases and controls in the same hospital or places, which could influence the results of study.

In conclusion, our results demonstrated the EBV DNA and VCA-IgA detection methods had better effect in diagnosis of NPC. However, EBV DNA detection method had high accuracy in diagnosis of NPC.
